# Impact of Tracheostomy on COVID-19 ICU Patients in Saudi Arabia: A Retrospective Analysis

**DOI:** 10.7759/cureus.52766

**Published:** 2024-01-22

**Authors:** Rawan A Alhazmi, Neeveen F Omer, Fatimah A Hameed, Sara Khan, Mohammed Khawajah, Hussain A Alabdullah, Tala O Althenayan, Amjad W Alhithlool, Ayman M Kharaba

**Affiliations:** 1 Medicine, Alrayan Medical Colleges, Madinah, SAU; 2 Medicine, Fakeeh College for Medical Sciences, Jeddah, SAU; 3 College of Medicine, Imam Abdulrahman Bin Faisal University, Dammam, SAU; 4 School of Medicine, Taibah University, Madinah, SAU; 5 Critical Care Medicine, King Fahad Hospital, Madinah, SAU

**Keywords:** future research directions, critical care strategies, regional healthcare variability, respiratory failure management, clinical practice implications, patient demographics analysis, tracheostomy outcomes, retrospective cohort study, saudi arabian healthcare, covid-19 icu tracheostomy

## Abstract

Introduction

The COVID-19 pandemic has prompted the development of novel medical interventions, including tracheostomy, a surgical procedure for a direct airway. This study investigates the intricacies of managing critically ill patients in the ICU, focusing on its debated utility in the global crisis.

Methods

The study assessed the impact of tracheostomy on COVID-19 patients at Al-Ahsa Hospital, Saudi Arabia, using a retrospective cohort design and data from electronic health records and databases. It aimed to provide insights into treatment outcomes and practices.

Results

The findings of this study shed light on the significant impact of tracheostomy on the course of ICU treatment for COVID-19 patients. Total number of participants were 1389. The study cohort consisted of predominantly non-pregnant individuals with an average body mass index reflective of the regional population. Among the COVID-19 patients, only a small percentage, 63 (4.5%), required tracheostomy, while the majority, 1326 (95.5%), did not undergo this procedure. Analysis of ICU outcomes revealed that a substantial proportion of patients, 223 (16.1%), achieved total cure, while the remaining patients did not. After a 28-day ICU stay, the majority of individuals, 1287 (92.7%), were discharged, while a smaller percentage remained in the ICU, with 77 (5.5%) still requiring mechanical ventilation. Notably, patients who underwent tracheostomy had a significantly longer ICU stay compared to those who did not, with an average of 59 days versus 19 days, respectively. Furthermore, the study found that tracheostomy did not significantly impact ICU discharge outcomes, including death, discharge home, and transfer to another facility. However, it did influence hospital discharge outcomes, with lower mortality rates and a higher rate of transfer to another facility among patients who underwent tracheostomy. These results provide valuable insights into the management and outcomes of critically ill COVID-19 patients in the ICU, particularly in relation to the use of tracheostomy as a treatment intervention.

Conclusion

The study highlights the dual benefits of tracheostomy in COVID-19 care, extending hospital stays but not increasing ICU discharge rates, emphasizing the need for tailored clinical strategies.

## Introduction

The advent of the COVID-19 pandemic has necessitated urgent and innovative approaches in the medical field, particularly concerning the management of critically ill patients. Among these approaches, tracheotomy, a surgical procedure to create a direct airway through an incision in the trachea, has been a pivotal intervention in the intensive care of patients with severe respiratory distress due to COVID-19. This study is positioned at the intersection of this critical intervention and its nuanced outcomes in a specific geographical and healthcare context. Tracheostomy's role in COVID-19 management has been the subject of extensive research. Earlier studies have delved into the varied outcomes of tracheostomy among COVID-19 patients, revealing a spectrum of benefits and challenges [1-3]. This variability in outcomes is influenced by factors such as the timing of the procedure, patient demographics, and underlying health conditions. The complexity of these factors is further underscored by systematic reviews and observational studies, which explore the timing and efficacy of tracheostomy in different patient cohorts [4,5]. Despite the breadth of global research, there remains a conspicuous gap in consolidated data focusing on the outcomes of tracheostomy among COVID-19 ICU patients in Saudi Arabia. This gap is noteworthy, considering the unique healthcare infrastructure and patient demographics of the region, which can significantly influence treatment outcomes, as indicated by the studies [6,7]. The varying healthcare systems, coupled with regional differences in patient populations, necessitate a tailored analysis of tracheostomy outcomes to inform local clinical practices effectively. Addressing this gap, the current study aims to retrospectively analyze the outcomes of tracheostomy in COVID-19 ICU patients in Saudi Arabia. The objectives are manifold and structured to ensure a comprehensive understanding: evaluating the efficacy and safety of tracheostomy in this specific patient group, analyzing patient data after the procedure, and assessing the implications for future treatment protocols. This retrospective analysis is not only specific and measurable but also achievable through the examination of existing medical records. It is highly relevant, given the ongoing pandemic and the evolving nature of COVID-19 treatment strategies, particularly in the context of Saudi Arabia. It promises to shed light on the effectiveness and risks associated with tracheostomy in a specific patient population, which is crucial for clinicians and healthcare policymakers in Saudi Arabia and potentially in similar healthcare contexts. As evidenced by further research, understanding the nuances of tracheostomy outcomes is critical for developing effective management strategies for severe COVID-19 cases [8,9]. The anticipated outcomes of this research are expected to contribute valuable insights into clinical practice, guiding the development of tailored treatment protocols and healthcare policies that are attuned to the specific needs of COVID-19 patients in ICU settings. 

## Materials and methods

Study design and setting

This retrospective cohort study was conducted to assess the impact of tracheostomy on COVID-19 patients in ICUs at Al-Ahsa Hospital, Saudi Arabia. The study timeframe extends from the beginning of the pandemic until 2022, focusing on historical patient data to provide insights into treatment outcomes and medical practices during this period. 

Participants

The study included adult COVID-19 patients who underwent tracheostomy at the ICUs of Al-Ahsa Hospital. 

Inclusion and exclusion criteria

Adult patients aged 18 or older, diagnosed with COVID-19 and admitted to the ICU primarily due to respiratory failure that necessitated tracheotomy as well as adults who are intubated for two weeks or more, are candidates for tracheostomy, were included. This criterion was in line with the study’s aim to evaluate outcomes after tracheostomy in this patient group. Excluded were individuals below 18 years, patients without a confirmed COVID-19 diagnosis, and those who underwent tracheostomy for non-COVID-19-related respiratory failure. This exclusion was vital to maintain the study’s focus on COVID-19-related respiratory complications. 

Data collection and measurement

Data were sourced from the electronic health records and ICU patient databases of Al-Ahsa Hospital. The variables measured included patient demographics, COVID-19 severity, comorbidities, tracheotomy procedure details, post-procedure complications, and outcomes (e.g., survival rates, length of ICU stay). This approach ensured data consistency across the study. 

Bias reduction and study size

Standardized data collection protocols and blinded data analysis were employed to minimize bias. The study’s sample size was derived from the available data at Al-Ahsa Hospital, ensuring statistical significance and robust conclusions. 

Materials and equipment

The primary materials were the electronic health record systems for data retrieval and SPSS version 28.0 (IBM Corp, Armonk, NY) for data analysis, facilitating the accurate handling of complex datasets. p-Values were calculated using chi-square.

Ethical considerations and data quality assurance

The study adhered to high ethical standards, with Institutional Review Board (IRB) approval obtained from King Faisal University (registration number 1702). Compliance with the Declaration of Helsinki was ensured, along with stringent patient confidentiality measures. Data integrity was maintained through meticulous cross-verification and regular audits. 

## Results

Demographic characteristics 

In Table 1, the demographic characteristics of the participants are detailed. The average age of the cohort was 56 years (N=1389, 100%), with a gender distribution of 361 females (26.0%) and 1028 males (74.0%). Of the female participants, 332 (94.3%) were not pregnant, while 20 (5.7%) were pregnant. The average BMI was 30.18±6.86. Regarding nationality, 693 patients (49.9%) were Saudi and 696 (50.1%) were non-Saudi. Healthcare workers comprised 65 individuals (4.7%). Only five patients (0.4%) had a history of travel outside Saudi Arabia. The majority of admissions were from home (1176, 84.7%), with only 3 (0.2%) from nursing homes and 210 (15.1%) transferred from other facilities.

**Table 1:** Demographic characteristics Reflects the distribution across age, gender, nationality, and other key demographics.

Variable	Options	Count	%
Age (years)		56±15	
Gender	Female	361	26.0
	Male	1028	74.0
If female, pregnant?	No	332	94.3
	Yes	20	5.7
BMI (kg/m^2^)		30.18±6.86	
Was patient Saudi or non-Saudi?	Non-Saudi	696	50.1
	Saudi	693	49.9
Healthcare worker	No	1324	95.3
	Yes	65	4.7
Did the case travel outside of Saudi?	No	1384	99.6
	Yes	5	0.4
Hospital admission source	Home	1176	84.7
	Nursing home	3	0.2
	Transfer from other facility	210	15.1

ICU and hospital length of stay 

Table 2 presents data on hospital and ICU length of stay (LOS) and mechanical ventilation (MV) duration. The mean hospital LOS was 21 days, with a standard deviation of 19 days. The mean ICU LOS was 14 days with a standard deviation of 14 days. The average MV duration was 9.89 days with a standard deviation of 13.47 days.

**Table 2:** ICU and hospital LOS ICU and hospital LOS represents the duration of hospital and ICU stays, as well as mechanical ventilation duration. LOS, length of stay.

	Mean	SD
Hospital LOS (days)	21	19
ICU LOS (days)	14	14
MV duration (days)	9.89	13.47

Tracheostomy therapy in COVID-19 patients admitted to the ICU

Table 3 focuses on tracheostomy therapy in COVID-19 patients admitted to the ICU. Out of the total sample, 63 patients (4.5%) underwent tracheostomy, while the majority, 1326 patients (95.5%), did not undergo this procedure.

**Table 3:** Tracheostomy therapy in COVID-19 patients admitted to the ICU. The occurrence of tracheostomy among ICU patients with COVID-19 has been evaluated.

Variable	Options	Count	%
Tracheostomy	No	1326	95.5
	Yes	63	4.5

Outcomes of ICU patients with COVID-19

Table 4 presents the outcomes of 1389 ICU patients with COVID-19, providing a detailed breakdown of the results. In terms of microbiological cure, 1166 patients (83.9%) did not achieve a cure, while 223 patients (16.1%) exhibited microbiological cures. At 28 days of ICU stay, 1287 patients (92.7%) were discharged from the ICU, 25 patients (1.8%) remained in the ICU without ventilation, and 77 patients (5.5%) were still in the ICU and ventilated. The ICU discharge outcomes indicated that 553 patients (39.8%) succumbed to the illness, 748 patients (53.9%) were discharged home, and 88 patients (6.3%) were transferred to another facility. Hospital discharge outcomes revealed 730 patients (52.6%) did not survive, 566 patients (40.7%) were discharged home alive, and 93 patients (6.7%) were transferred to another facility.

**Table 4:** Outcomes of ICU patients with COVID-19 Outcomes of ICU patients with COVID-19 reveal the distribution of outcomes such as microbiological cure, ICU discharge status, and hospital discharge status.

Variable	Options		Count	%
Microbiological cure (defined as 2 consecutive samples negative COVID-19 test)		No	1166	83.9
		Yes	223	16.1
28 days of ICU stay		Discharged from ICU	1287	92.7
		Still in ICU, not ventilated	25	1.8
		Still in ICU, ventilated	77	5.5
ICU discharge outcome		Death	553	39.8
		Discharge home	748	53.9
		Transfer to another facility	88	6.3
Hospital discharge outcome		Death	730	52.6
		Discharge home alive	566	40.7
		Transfer to another facility	93	6.7

Association between tracheostomy and hospital and ICU LOS 

Table 5 explores the association between tracheostomy and hospital and ICU LOS. Patients without tracheostomy had a mean ICU LOS of 19 days (SD=16), while those with tracheostomy had a significantly longer mean ICU LOS of 59 days (SD=31, p<0.001). Similarly, patients without tracheostomy had a mean hospital LOS of 12 days (SD=11), while those with tracheostomy had a longer mean hospital LOS of 45 days (SD=26, p<0.001). The mean MV duration was 8.47 days (SD=10.97) for patients without tracheostomy and 33.60 days (SD=24.75) for those with tracheostomy (p<0.001).

**Table 5:** Association between tracheostomy and hospital and ICU LOS Association between tracheostomy and hospital and ICU LOS was evaluated using Mann-Whitney test.

	Tracheostomy				p-Value
	No		Yes		
	Mean	SD	Mean	SD	
ICU LOS (days)	19	16	59	31	<0.001
Hospital LOS (days)	12	11	45	26	<0.001
MV duration (days)	8.47	10.97	33.60	24.75	<0.001

Effect of tracheostomy on ICU outcomes of patients with COVID-19

Table 6 investigates the effect of tracheostomy on ICU outcomes in COVID-19 patients. Patients with tracheostomy had a lower rate of microbiological cure (30.2% vs. 86.5%, p<0.001) and a higher proportion still in the ICU at 28 days, either ventilated (60.3% vs. 2.9%, p<0.001) or not ventilated (3.2% vs. 1.7%, p<0.001). However, tracheostomy did not significantly impact ICU discharge outcomes, including death (42.9% vs. 39.7%, p=0.231), discharge home (47.6% vs. 54.1%), and transfer to another facility (9.5% vs. 6.2%). Tracheostomy significantly influenced hospital discharge outcomes, with a lower mortality rate (39.7% vs. 53.2%, p=0.003) and a higher rate of transfer to another facility (15.9% vs. 6.3%). 

**Table 6:** Effect of tracheostomy on ICU outcomes of patients with COVID-19

		Tracheostomy				p-Value
		No		Yes		
		Count	N %	Count	N %	
Microbiological cure (defined as 2 consecutive samples negative COVID-19 test)	No	1147	86.5	19	30.2	<0.001
	Yes	179	13.5	44	69.8	
28 days of ICU stay	Discharged from ICU	1264	95.3	23	36.5	<0.001
	Still in ICU, not ventilated	23	1.7	2	3.2	
	Still in ICU, ventilated	39	2.9	38	60.3	
ICU discharge outcome	Death	526	39.7	27	42.9	0.231
	Discharge home	718	54.1	30	47.6	
	Transfer to another facility	82	6.2	6	9.5	
Hospital discharge outcome	Death	705	53.2	25	39.7	0.003
	Discharge home alive	538	40.6	28	44.4	
	Transfer to another facility	83	6.3	10	15.9	

The relatively low incidence of tracheostomy underscores its selective use in this population. Patients undergoing tracheostomy exhibited significantly longer ICU and hospital stays, as well as increased MV duration. Furthermore, tracheostomy was associated with lower rates of microbiological cure but did not significantly impact ICU discharge outcomes. These findings contribute valuable insights into the clinical course and implications of tracheostomy in the management of severe COVID-19 cases. 

## Discussion

Our investigation delves into the implications of tracheostomy among ICU patients with COVID-19 within the distinctive healthcare milieu of Saudi Arabia, resonating with our research aims and employing a retrospective cohort framework akin to that used in previous studies [10]. The retrospective design, while offering a comprehensive overview, is not without the customary limitations associated with such approaches, including the potential for selection bias and reliance on the accuracy of existing records. 

The study's revelations, particularly that tracheostomy is associated with prolonged ICU and hospital stays (mean ICU LOS for tracheostomized patients was 59 days compared to 19 days for non-tracheostomized patients, p<0.001), and a significantly extended duration of MV (mean MV duration for tracheostomized patients was 33.60 days vs. 8.47 days, p<0.001), paint a nuanced picture of the tracheostomy's role in severe COVID-19 cases. These findings align with the spectrum of outcomes noted in other regional studies [11,12] and are an addition to the body of evidence suggesting that tracheostomy may be beneficial under certain clinical conditions. Noteworthy is the fact that despite these extended durations, tracheostomy did not markedly affect ICU discharge outcomes such as mortality or transfer rates when compared to non-tracheostomized patients. 

Individuals who need a feeding tube, tracheostomy, or both are typically sicker than those who do not. Despite this, when the data were sorted according to the quantity of CCCs (complex chronic conditions), owning a feeding tube was linked to a reduced three-day mortality overall and a lower 30-day mortality. Having both devices was linked to a decreased three-day mortality rate in patients who were admitted for urgent or emergent care as well as low-risk surgery [13].

Our data accentuate the influence of regional practice variations and patient demographics on treatment outcomes, a facet that is often underrepresented in the literature [14-16]. This underscores the necessity for region-specific research to fully grasp the intricacies of global health challenges. 

The timing of tracheostomy procedures remains a pivotal factor in patient outcomes, consistent with previous guidelines and considerations [17]. The observed outcomes in the Saudi context add empirical weight to these guidelines and reflect larger trends reported in broader analyses and systematic reviews [18,19]. 

While the findings are immediately relevant to the Saudi Arabian healthcare framework, their implications can be extrapolated to an international setting, contributing to the global dialogue on the management of severe COVID-19 cases as evidenced by comparisons with global data [20]. However, we must exercise caution in generalizing these results due to the retrospective and region-centric nature of the study. 

Acknowledging the limitations inherent in our retrospective analysis is crucial, as these may affect the interpretation of our findings and their application in clinical settings. Future research should, therefore, pivot toward prospective studies involving more diverse patient cohorts to validate and potentially universalize the role of tracheostomy in COVID-19 treatment strategies. This could fortify the development of standardized treatment protocols. Alternative research methodologies, such as randomized controlled trials, could yield more concrete evidence regarding the effectiveness and safety of tracheostomy for COVID-19 patients. 

Our research makes a significant addition to the compendium of knowledge regarding COVID-19 management, particularly pertaining to tracheostomy in ICU contexts. It extends beyond the Saudi Arabian healthcare system, providing insights that may inform clinical practices and health policy in analogous international settings. As the fight against COVID-19 continues to evolve, our study underscores the enduring need for ongoing research into the optimal treatment of severe cases of this disease. 

## Conclusions

The results not only exhibit varied patient outcomes that underscore the necessity for regionally tailored medical protocols but also signal the potential for significant advancements in clinical practices and health policy. By delving into the interplay between local healthcare infrastructure and patient demographics, this work contributes a pivotal piece to the broader puzzle of global health management amid a pandemic. Such insights advocate for a flexible, culturally aware approach to health crises, setting the stage for further scholarly inquiry and the strategic evolution of treatment guidelines across diverse medical ecosystems. 
